# A Rare Malignant Case of a Primary Pseudomyogenic Haemangioendothelioma of the Bone

**DOI:** 10.3390/curroncol32040219

**Published:** 2025-04-10

**Authors:** Annabella Di Mauro, Salvatore Tafuto, Lucia Cannella, Francesca Collina, Giovanni Neri, Ottavia Clemente, Imma D’Arbitrio, Francesca Ricci, Secondo Lastoria, Gerardo Ferrara, Annarosaria De Chiara

**Affiliations:** 1 Pathology Unit, Istituto Nazionale Tumori, IRCCS—Fondazione G. Pascale, 80131 Napoli, Italy; annabella.dimauro@istitutotumori.na.it (A.D.M.); francesca.collina@istitutotumori.na.it (F.C.); immadarbitrio@istitutotumori.na.it (I.D.); gerardo.ferrara@istitutotumori.na.it (G.F.); 2 Sarcomas and Rare Tumors Unit, Istituto Nazionale Tumori, IRCCS—Fondazione G. Pascale, 80131 Naples, Italy; s.tafuto@istitutotumori.na.it (S.T.); giovanni.neri@istitutotumori.na.it (G.N.); ottavia.clemente@istitutotumori.na.it (O.C.); 3 Nuclear Medicine Unit, Istituto Nazionale Tumori di Napoli, IRCCS G. Pascale, 80031, Italy; francesca.ricci@istitutotumori.na.it (F.R.); s.lastoria@istitutotumori.na.it (S.L.); 4 Histopathology of Lymphomas and Sarcomas SSD, Istituto Nazionale Tumori, IRCCS—Fondazione G. Pascale, 80131, Naples, Italy; a.dechiara@istitutumori.na.it

**Keywords:** bone tumors, rare tumors, primary pseudomyogenic haemangioendothelioma of the bone, diagnosis, pathology, whole-body imaging, cancer metastasis

## Abstract

Pseudomyogenic haemangioendotheliomas (PMH) are exceedingly rare, mostly occurring in soft tissue, with malignant cases even more uncommon. In this report, we present a case of a 28-year-old male initially suspected of having a fibroblastic osteosarcoma of the right femur, which was then correctly diagnosed as a primary pseudomyogenic hemangioendothelioma of the bone with synchronous metastases to other skeletal segments. Molecular analysis through targeted RNA sequencing confirmed the correct diagnosis, revealing a fusion transcript *ACTB::FOSB*. To our knowledge, this is one of the few reported cases of suffering from multiple pathological fractures. The rapid skeletal progression and the onset of distant metastases in this case is highly unusual considering the typically indolent clinical course commonly reported in the literature for this tumor.

## 1. Introduction

Pseudomyogenic haemangioendothelioma (PMH) is a rare, mostly indolent, low-grade vascular neoplasm that often histologically mimics myoid and epithelioid tumours. Although this tumour lacks histological features of vascular differentiation, it retains some cytological features and immunohistochemical expression of endothelial differentiation markers, such as CD31 and ERG. This entity was first recognized in 2003 by Bilings et al. [1] as a low-grade vascular tumour mimicking an epithelial sarcoma, being initially named epithelioid sarcoma-like haemangioendothelioma. None of patients included in the original group had distant metastases. The term pseudomyogenic hemangioendothelioma (PMH) was introduced in 2011 by Hornik et al. [2], and in 2013 this terminology was adopted by the World Health Organisation’s. It is most frequent in young adult males, and usually presents as multiple nodules on the skin, most often localised to the extremities. It can also involve several tissue planes simultaneously and may affect the dermis, subcutis, skeletal muscle and bone [3,4,5]. In the bone, on radiological examination, it is usually well circumscribed and has a lytic appearance [6]. Histologically, it is characterised by a nodular pattern composed of fascicles and sheets of spindle cells with vesicular nuclei, nucleoli of variable size, and abundant eosinophilic cytoplasm. The cells are epithelioid with “pseudomyogenic” morphology. There is no histological evidence of vascular growth, although some cells show intracytoplasmic vacuolization, suggesting vascular differentiation. This can be demonstrated by the immunohistochemical expression of CD31, CD34, ERG and FLI1. Epithelioid cells may express cytokeratins and epithelial markers, such as AE1/AE3, EMA and P63 [3,4,7,8]. After the initial discovery of a balanced t (7;19) (q22; q13) translocation in two cases [9], subsequent RNA sequencing identified first a *SERPINE1* to *FOSB* fusion [10]. Recently, novel fusions of the *FOSB* gene with *ACTB* (7p22) and *WWTR1* (3q25) [11,12] have been described, all resulting in increased *FOSB* protein expression and immunohistochemical detection. Sugita et al. [13] and Hung et al. [14] investigated the immunostaining of FOSB and CAMTA1 in vascular carcinomas and found that the immunostaining of FOSB was very sensitive and specific for PMH [15]. We present an unusually aggressive case of primary bone PMH with malignant behaviour.

## 2. Case Presentation

**Figure 1:**
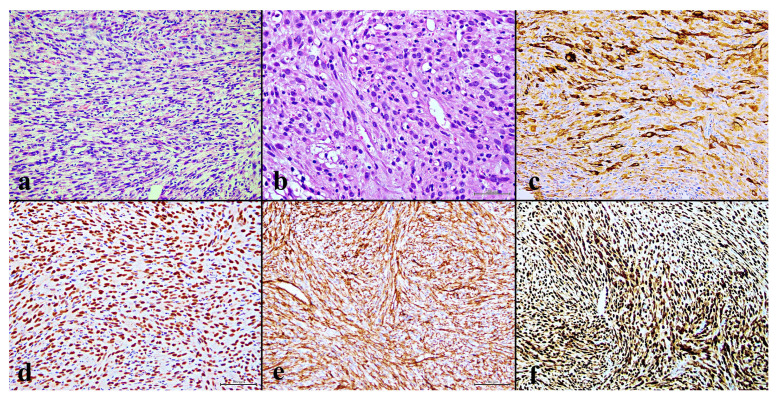
(**a**) Fascicles of spindle cells with eosinophilic cytoplasm are visible (H&E, 20×); (**b**) scattered spindle-shaped tumor cells with intracytoplasmic vacuoles suggest their vascular origin (H&E, 20×); (**c**) a mixture of epithelioid tumor cells that are positive for CK AE1/AE3 (20×); (**d**) nuclear expression of ERG; (**e**) diffuse membrane expression of CD31 (20×); (**f**) and strong nuclear expression of FOSB (20×).

In September 2022, a 28-year-old male presented to our institute following a multifragmentary diaphyseal fracture of the right femur. A previous biopsy conducted at an external institution suggested a diagnosis of osteosarcoma. Imaging revealed an osteolytic lesion (6 × 2 cm) in the left scapula and osteothickening areas in thoracic vertebrae T9-T11, L2, both iliac wings, the right ninth rib, and the ipsilateral femoral head. The biopsy slides were reviewed at our institution, revealing mostly bone “callus” related to the fracture, but a small fragment strongly suggested Pseudomyogenic Haemangioendothelioma (PMH). A new biopsy taken from the left scapula was carried out at our institution. Histology again showed the hypercellular mesenchymal proliferation of elongated cells with eosinophilic cytoplasm and focal necrosis, and no evidence of an osteoid matrix. The tumor cells showed strong diffuse staining for CD31, ERG and FOSB, and negative on CD34 and CAMTA, with a minority of TFE3, CKAE1/AE3+ and EMA [Figure 1].

A targeted NGS panel of 55 genes sarcoma-specific (Archer FusionPlex Expanded Sarcoma Panel v. 1.5 (Invitae, Boulder, CO, USA)), was used to detect the presence of the fusion transcript *ACTB* (exon3):: *FOSB* (exon2) (NM_00101. 3: NM_006732.2), with the following main breakpoint predicted: chr7:5568792, chr19:45973887. The combined morphoimmunological features and molecular studies led to a diagnosis of a primary bone PMH.

**Figure 2:**
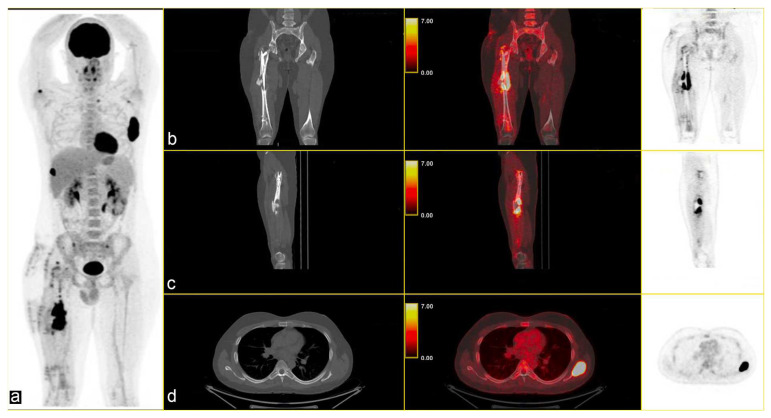
(**a**) Maximum Intensity Projection (MIP) shows several skeletal lesions at different sites; CT-PET fusion images: (**b**,**c**) sagittal images of the primary lesion in the left femur and (**d**) an osteolytic lesion (SUV_max_ 35) in the left scapula (**b**–**d**): CT on the left, CT fused images in the middle, and PET image on the right.

The multidisciplinary tumour committee recommended positron emission tomography (PET), which revealed multiple metabolically active lytic/sclerotic lesions in several bone sites: the right femur (SUV_max_ 22.5) at the level of the lateral margin of the left scapula (SUV_max_ 35), the glenoid cavity of the ipsilateral scapula (SUV_max_ max 6.7), the right humerus (SUV_max_ 10.1), in the lateral side of the right rib (SUV_max_ 22), in the left transverse process D1 (SUV_max_ 5.4), and in correspondence with the thoracic vertebrae T11 (SUV_max_ 5.4) [Figure 2].

Treatment with a chemotherapy (CHT) regimen was chosen. First-line chemotherapy with epirubicin 60 mg/mq on days 1 and 2, and ifosfamide 3000 mg/mq on days 1, 2 and 3 every 3 weeks, was started. The patient was re-evaluated by a FDG PET/CT only after seven months, due to an allergy to the iodinated contrast medium.

Anti-cancer therapy was followed for a total of five cycles, achieving metabolic stability of the disease, but several side effects were observed, leading to therapy discontinuation. The adverse events included G4 toxicity and G3 anaemia. Specifically, the G4 toxicity consisted of persistent G4 neutropenia and G4 thrombocytopenia during the long course of treatment.

**Figure 3:**
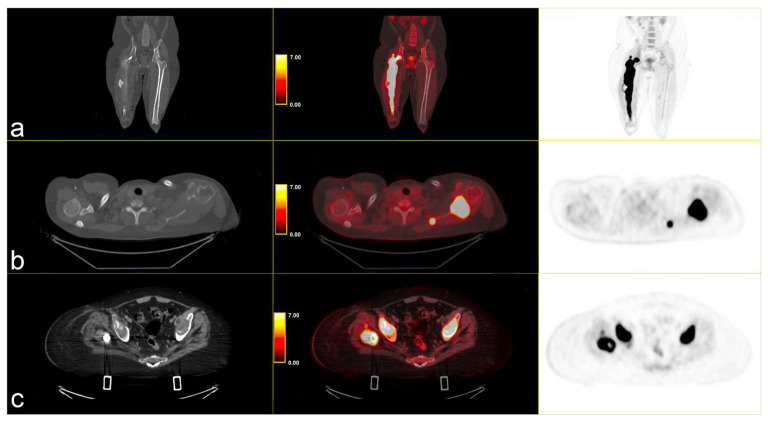
PET/CT fused images show large hypermetabolic lesions in (**a**) the right femur, (**b**) the left scapula (with erosion of the cortical part), (**c**) thickened osteogenic lesions in the pelvis and bilateral iliac wings (thickened osteogenic lesions in with lower SUVmax: 6.7). From left to right CT PET/CT fused images and PET images.

Following instrumental and clinical disease progression, the patient was treated with Sirolimus (an m-TOR inhibitor). At the first re-evaluation, instrumental progressive disease (PD) was observed with a moderate increase in bone SUV_max_, particularly in the left scapula (SUV_max_ 34.8 vs. 31), and by the appearance of new lesions, such as the right iliac wing, numerous pulmonary nodules (SUV max 6) and lymph nodes in the left axilla (SUV_max_ 6.1) [Figure 3].

**Figure 4:**
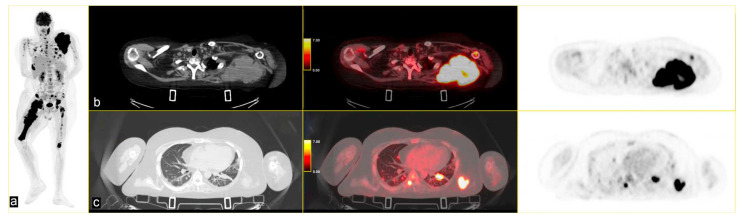
(**a**) MIP PET/CT showing significant disease progression; (**b**) volumetric and metabolic increase of the lesion in the left scapula (SUV_max_ 35); and (**c**) multiple lesions in the upper lobes of both lungs (SUVmax 25.3) on (**b**,**c**) strips CT on the left, PET/CT fused images the middle and PET images on the right.

The patient continued third-line antineoplastic treatment with Gemcitabine (750 mg/mq) and Taxotere (75 mg/mq) for three cycles until disease progression. Fourth-line treatment with trabectedin (1.25 mg/m^2^) followed, reporting bone marrow and liver toxicity. The last PET scan, performed in May 2024, showed an increased uptake of FDG in several pseudonodular lesions in both lungs at the pleuro-parenchymal level (SUV_max_ 25.3 vs. 10.9), and in the left axillary lymphnodes (SUV_max_ 17.4 vs. 6.1). A new, unknown area of glucose hypermetabolism was depicted in the body of the pancreas (SUV_max_ 4.0), highly suspicious for metastasis. The lesion of the right femur extended to the whole bone, with other secondary lesions in the skull [Figure 4].

A progressive clinical deterioration was observed, which led to his confinement to bed and to analgesic therapy until his death in July 2024.

## 3. Discussion

The current World Health Organisation (WHO) classification of soft tissue and bone tumors places Pseudomyogenic haemangioendothelioma (PMH) in the group of rarely metastasising vascular neoplasms with an intermediate malignant potential [15]. Common sites of PMH appearance are the dermis and hypodermis, but about half of cases originate from the muscles and 20% from the skeleton [4,6,8,9]. The histological morphology of PMH consists of enlarged, spindled neoplastic cells with bright eosinophilic cytoplasm, mimicking rhabdomyoblasts. They contain a low level of nuclear atypia and rare mitotic activity [3,5]. PMH has an inclusive differential diagnosis of epithelioid sarcoma, rhabdomyosarcoma, osteoblastoma and vascular tumors [14]. PMH presents similarities with epithelioid sarcomas, such as presenting in the skin and soft tissue in the distal extremities, and having diffuse keratin positivity [1]. Due to the extreme rarity of this vascular tumor and its less characteristic morphological features, the histological diagnosis of PMH is sometimes difficult. It is well established that PMH is immunohistochemically positive for the keratins AE1/AE3 and the endothelial transcription factor ERG, as well as CD31 and CD33 [1,3,4]. The WHO 2020 classification recently reported genetic alterations in PMH [15]. Fusion transcripts involving the *FOSB* gene on 19q13 with *SERPINE1* (7q22), *ACTB* (7p22) and *WWTR1* (3q25) have been detected and may be useful in the differentiation of PMH from other vascular tumors. This genetic alteration (t(7;19) (q22;q13;q25)) is considered to be both the causative and a useful diagnostic marker of PMH, and FOSB positivity on immunohistochemistry is an excellent surrogate marker for the presence of this genetic alteration [10,11,12,13,14]. In the present case, immunohistochemical positivity for FOSB, together with CD31 and ERG, and NGS analysis, highlights the *ACTB::FOSB* fusion transcript, and led to the diagnosis of PMH. The detection of *ACTB::FOSB* fusion is consistent with PMH’s diagnosis and supports its differentiation from other sarcomas, with both diagnostic and potential therapeutic implications. This fusion transcript is known to promote tumorigenesis by dysregulating FOSB, a gene involved in proliferating and differentiating cells [13,14]. The optimal treatment strategy for PMH is still undefined. Patients were treated with surgery or chemotherapy or both. Only three patients (5%) had metastatic disease, detected 4, 8, 5 and 16 years after initial diagnosis. Recent trials of everolimus or a combination of gemcitabine and docetaxel have shown convincing results, with some reduction in tumor size, but further large studies may clarify the precise effectiveness [5,7,16,17,18,19]. In our case, the patient was treated with chemotherapy. The EPI/IFO (epirubicin and ifosfamide) regimen was administered for four cycles over three weeks [18,20,21]. The EPI/IFO, gemcitabine and docetaxel, trabectedin and m-TOR inhibitor obtained modest disease control and mild–severe hematological toxicity. To date, only two patients have died as a result of the disease. One patient, a 19-year-old female, died after 201 months of follow up [22]. In our case, after two years, the patient developed metastases in the lungs and progressive bone involvement, with an unfortunate outcome. This case report highlights the importance of a multidisciplinary approach and the usefulness of molecular diagnostics in confirming the diagnosis of rare tumors like PMH, especially in atypical presentations. As far as we are concerned, we present one of the few cases [22,23] of unusually aggressive PMH originating from bone. While PMH is generally classified as an intermediate-grade endothelial tumor, in rare cases it can exhibit highly aggressive clinical behavior, as observed in our patient. This underscores the importance of considering both histologic and clinical features when characterizing these tumors. The variable clinical outcome of PMH highlights the need for further studies to elucidate prognostic factors and optimize treatment strategies for patients with aggressive/metastatic disease.

## 4. Material and Methods

RNA was extracted from FFPE tumour tissue using the RNeasy FFPE Kit (Qiagen, Hilden, Germany) according to the manufacturer’s protocol. A commercially available NGS-based assay that analyses 55 genes involved in sarcoma-related alterations (Archer FusionPlex Expanded Sarcoma Panel v. 1.5 (Invitae, Boulder, CO, USA), based on Anchored Multiplex PCR (AMP) chemistry, was used to detect gene fusions with known and unknown fusion partners, selected hotspot single nucleotide variants (SNVs), indels, splicing defects and exon skipping. Sequencing was performed on an Ion GeneStudio S5 System-Ion Torrent platform (Thermo Fisher Scientific, Waltham, MA, USA). The sequencing data were analysed with Archer Analysis software v. 6.2 (Invitae, Boulder, CO, USA).

## 5. Conclusions

Pseudomyogenic haemangioendothelioma (PMH) is a rare vascular tumor that primarily affects young adults. While PMH typically follows a more indolent course with a low risk of metastasis, our case represents an unusually aggressive form of disease with a fatal outcome. Molecular biology, in conjunction with an appropriate IHC panel, is an essential diagnostic tool for confirmation of the diagnosis. This case highlights the diagnostic challenges in identifying primary PMH and its rapid skeletal progression. It provides insight into its atypical clinical behaviour and highlights the importance of a multidisciplinary diagnostic approach, as well as the need for novel therapeutic approaches to manage aggressive or refractory cases.

## Data Availability

Data are contained within the article.
